# Self-perceived digital literacy, creative self-efficacy, and self-reported innovative behavior among undergraduate nursing students in Anhui, China: a multi-institutional cross-sectional study

**DOI:** 10.3389/fpubh.2026.1938122

**Published:** 2026-09-30

**Authors:** Tong Wu, Xudong Fan, Xinle Zhou, Yingying Wang, Miao Wang

**Affiliations:** 1 School of Nursing, Wannan Medical University, Wuhu, China; 2 Department of Emergency, Yijishan Hospital of Wannan Medical University, Wuhu, China

**Keywords:** creative self-efficacy, digital health workforce, digital literacy, innovative behavior, nursing education, undergraduate nursing students

## Abstract

**Background:**

Nursing studies have separately linked self-perceived digital literacy with self-reported innovative behavior and the clinical practice environment with creative self-efficacy and innovative behavior. A related association pattern has been reported among Chinese medical students, but joint evidence from multi-institutional nursing samples remains limited.

**Objective:**

To evaluate the measurement structure and examine the three-variable association pattern among self-perceived general digital literacy, creative self-efficacy, and self-reported innovative behavior in nursing students recruited from three institutions.

**Methods:**

A multi-institutional cross-sectional online survey conducted from September to December 2025 included 502 undergraduate nursing students from three medical higher-education institutions in Anhui Province, China. Item-level confirmatory factor analyses compared five theoretically plausible measurement models, and convergent and discriminant validity were evaluated before PROCESS Model 4 was applied to observed total scores. The primary analysis used 5,000 bootstrap resamples and eight theory-informed covariates.

**Results:**

Self-perceived general digital literacy was correlated with creative self-efficacy (*r* = 0.574) and self-reported innovative behavior (*r* = 0.584), while creative self-efficacy was strongly correlated with self-reported innovative behavior (*r* = 0.834; all *p* < 0.001). The theory-consistent higher-order three-construct model showed acceptable fit (*χ*^2^ = 1314.15, df = 426, CFI = 0.935, TLI = 0.929, RMSEA = 0.065, SRMR = 0.068), fitted substantially better than the one-factor model, and significantly outperformed the corresponding model combining creative self-efficacy and innovative behavior (Δ*χ*^2^ = 151.76, Δdf = 2, *p* < 0.001). Their latent correlation was 0.907, HTMT was 0.902 (95% CI, 0.858–0.941), and the Fornell-Larcker criterion was not met. In the adjusted observed-score model, the total coefficient, direct coefficient, and bootstrap product term involving creative self-efficacy were 0.145 (95% CI, 0.127–0.163), 0.040 (95% CI, 0.025–0.055), and 0.105 (95% CI, 0.087–0.124), respectively. The product-term confidence interval excluded zero, and its estimate was materially similar without covariate adjustment. A supplementary *post hoc* reparameterization based on the observed-score correlations indicated that the data did not uniquely determine the directional ordering.

**Conclusion:**

In this pooled three-institution nursing-student sample, the three self-report constructs showed a coherent cross-sectional association pattern across measurement-model comparisons and adjusted and unadjusted specifications. This finding connects two previously separate lines of nursing evidence and extends the complete pattern beyond a single-campus nursing sample. Creative self-efficacy was a theoretically relevant correlate within the contemporaneous statistical decomposition, not an established temporal or causal mechanism. Longitudinal studies with objective or observer-rated measures are needed to test temporal ordering and educational or practice-relevant implications.

## 1 Introduction

Digital technologies increasingly shape clinical care, public health practice, health information management, interprofessional communication, and professional education. The World Health Organization’s Global Strategy on Digital Health 2020–2027 emphasizes the development of human and organizational capacity for safe, effective, and equitable digital transformation, and a recent WHO landscape analysis identifies competency frameworks as an important basis for health workforce education and practice (1, 2). China’s 14th Five-Year Plan for National Health similarly prioritizes digital transformation, workforce development, and health-service innovation (3). Undergraduate nursing students are therefore entering a profession in which the ability to use digital information critically and responsibly is relevant to academic learning and future practice across clinical, community, and public health settings. In this study, digital literacy was operationalized as students’ self-perceived general ability to locate, evaluate, create, communicate, and apply information in digital environments. This broad educational construct provides insight into students’ perceived digital resources for learning, but it is distinct from objectively demonstrated digital competence, digital health literacy, nursing informatics proficiency, and performance in clinical digital systems (4).

Innovative behavior is commonly conceptualized as the generation, championing, and implementation of ideas intended to improve work processes or outcomes (5). The present study operationalized this construct using a self-report scale that captures students’ reported tendencies to generate, promote, and apply ideas. Such tendencies are educationally relevant antecedent-oriented indicators, although they are not equivalent to independently verified implementation or downstream practice outcomes. Self-perceived digital literacy may be associated with these tendencies because locating and appraising evidence, integrating information, communicating across sources, and creating digital content can support the development and articulation of new ideas. Cross-sectional research has characterized self-perceived digital competencies among nursing students (6), and a recent latent-profile study of Chinese undergraduate nursing students found that students with higher self-reported digital-literacy profiles also reported more innovative behavior (7). Evidence from medical students similarly links digital-literacy configurations with innovative behavior (8). These findings support the digital literacy-innovative behavior segment of the proposed model across nursing and medical education, but they do not determine whether creative self-efficacy is simultaneously involved among nursing students.

Social cognitive theory proposes reciprocal interactions among personal factors, behavior, and the environment, with self-efficacy shaping choice, effort, persistence, and responses to setbacks (9). Creative self-efficacy is the domain-specific belief that one can produce creative outcomes (10). It was selected as the focal theory-informed construct in the proposed statistical ordering because it is conceptually distinct from both perceived digital resources and reported innovative action, yet is theoretically proximal to idea-focused behavior. Self-perceived digital literacy may reflect the information, communication, content-creation, and problem-solving resources that students believe they can draw on; however, perceiving such resources does not by itself imply that a student will generate, champion, or apply a new idea. Creative self-efficacy concerns a different and more task-proximal appraisal: whether the student believes that available knowledge and skills can be mobilized successfully in a creative task. Such beliefs are relevant to selecting challenging idea-related activities, sustaining effort under uncertainty, and persisting after feedback or setbacks (9, 10). Thus, creative self-efficacy is positioned as a domain-specific efficacy appraisal that may help characterize the association between perceived digital capability and self-reported innovative behavior, rather than as a proxy for either construct or as a mechanism established by the cross-sectional design.

The relevance of this theoretical distinction to nursing education is supported by evidence from clinical learning contexts. Xiang et al. reported that a more positive clinical practice environment was associated with greater creative self-efficacy and more innovative behavior among nursing students, with a statistically significant indirect association involving creative self-efficacy (11). That study positions creative self-efficacy as a plausible correlate within a nursing-student innovation model, but it did not include digital literacy. Conversely, a single-university cross-sectional study of 887 Chinese medical students reported an indirect path from digital literacy to innovative behavior involving creative self-efficacy within a broader chain model that also included diversity experience (12). Taken together, social cognitive theory and these complementary empirical findings provide a parsimonious rationale for examining a product-of-coefficients component involving creative self-efficacy in the proposed statistical ordering, while leaving alternative cognitive or motivational explanations open. The unresolved issue is whether all three focal constructs are jointly associated in nursing students recruited from multiple institutions.

This study was designed to address that specific gap rather than to claim first identification of the association pattern. The objective was to evaluate the measurement structure and examine the contemporaneous relationships among self-perceived general digital literacy, creative self-efficacy, and self-reported innovative behavior in undergraduate nursing students recruited from three institutions in Anhui Province, China. Recruitment from three institutions broadened the institutional sources represented and allowed assessment of whether the association pattern was observable in a pooled nursing sample that was not drawn entirely from one campus. Because institution-specific effects were not estimated, the design does not establish consistency of the associations across the three institutions. The study also did not directly compare nursing and medical students and therefore was not designed to test profession-level differences. Before interpreting observed-score associations, we assessed whether item-level responses supported retaining the three constructs relative to plausible models that combined creative self-efficacy with innovative behavior or placed all items on one factor. We then examined whether (1) self-perceived general digital literacy was associated with self-reported innovative behavior, (2) self-perceived general digital literacy was associated with creative self-efficacy and creative self-efficacy was associated with self-reported innovative behavior, and (3) the observed total-score data contained a nonzero product-of-coefficients component involving creative self-efficacy. The primary observed-score analysis adjusted for a theory-informed set of demographic and educational covariates, and the unadjusted model was retained as a sensitivity analysis. Accordingly, the study evaluates a cross-sectional pattern among student-reported perceptions, efficacy beliefs, and behavioral tendencies rather than objective performance, temporal order, or causal mediation.

## 2 Materials and methods

### 2.1 Study design, setting, and reporting

A multi-institutional cross-sectional online survey was conducted from September to December 2025 at three medical higher-education institutions in Anhui Province, China. Recruiting across three institutions broadened the institutional sources represented within the province compared with a single-campus study and provided a pooled setting in which the proposed association pattern could be evaluated. Participants were recruited by convenience sampling. Because this non-probability approach did not yield known inclusion probabilities, inference was limited to the pooled sample rather than all undergraduate nursing students in Anhui Province. This report was prepared with reference to the Strengthening the Reporting of Observational Studies in Epidemiology (STROBE) statement and the Checklist for Reporting Results of Internet E-Surveys (CHERRIES) (13, 14). Relevant CHERRIES items—including the online access and recruitment context, voluntariness and electronic consent, survey duration, completeness and logic checks, duplicate-entry safeguards, eligibility screening, handling of incomplete or atypical records, and the response metrics that were available or could not be reconstructed—are reported in Sections 2.2 and 2.4 and in the participant-flow results.

### 2.2 Participants and sample-size planning

Full-time undergraduate nursing students enrolled at the participating institutions were eligible if they voluntarily agreed to participate and provided electronic informed consent. Three exclusion criteria—being away from campus because of academic leave, medical leave, or another reason; reporting a major life event during the preceding 3 months; and self-reporting a mental or psychological disorder—were included in the protocol reviewed and approved by the Ethics Committee. Eligibility was assessed by self-report during online screening. “Major life event” was not operationalized through a standardized event list, severity threshold, or validated instrument and therefore depended on the student’s own interpretation. “Mental or psychological disorder” referred to self-reported status and was not verified by a study-administered diagnostic assessment. No eligibility-screening log or reason-specific count is available for this report, and the final analytic dataset contains only eligible respondents. Accordingly, the number excluded for each criterion and any overlap between criteria cannot be reconstructed.

The protocol used a transparent two-stage pragmatic approach to recruitment planning and set a minimum target of 275 participants. First, it applied the commonly cited heuristic of approximately 10 respondents per item in scale-development and validation work to the 22-item primary digital-literacy measure, yielding an analyzable-sample benchmark of 220 participants (15). This convention was used as an operational benchmark, not as an effect-size-based calculation for the product-of-coefficients term. Second, to preserve 220 analyzable records under a prospectively specified 20% allowance for submitted records that might be incomplete or fail prespecified quality-control criteria, the recruitment target was calculated as 220/(1–0.20) = 275 (16). The exact 20% was an investigator-specified planning allowance; the available study documentation did not identify a pilot estimate or site-specific historical loss rate used to derive it, and it is not presented as a universal recommendation. The final analytic sample of 502 substantially exceeded the target by 82.5% and corresponded to 22.8 respondents per item for the 22-item primary measure. As an analysis-specific retrospective benchmark, published simulations for a simple mediation model indicate that a percentile-bootstrap test requires approximately 558 participants for 80% power when both standardized component paths are small (0.14), 414 when one path is small (0.14) and the other small-to-medium (0.26), and 78 when both paths are medium (0.39) (17). Thus, the achieved sample was close to the conservative small-small benchmark and exceeded the other two scenarios. The observed standardized component paths were 0.565 and 0.740, both above the simulated medium-path value. Because these paths were estimated from the analyzed data, the comparison is a retrospective sensitivity analysis rather than an *a priori* power calculation. Precision of the principal product-term estimate was evaluated directly from its 5,000-resample bootstrap 95% CI. Together, the achieved sample, simulation benchmarks, and bootstrap interval provide complementary evidence on the sensitivity and empirical precision of the primary product-of-coefficients analysis without claiming prospectively determined precision for every regression or confirmatory-factor-analysis parameter.

### 2.3 Measures

#### 2.3.1 Demographic and educational characteristics

A researcher-developed questionnaire collected demographic and educational information, including sex, year of study, rural or urban residence, only-child status, parental educational attainment, household income, reason for choosing nursing, student leadership experience, research-project participation, competition awards, scholarships, proficiency certificates, and participation in extracurricular practice activities.

#### 2.3.2 Digital literacy

Digital literacy was assessed with a 22-item Chinese questionnaire developed by Liu for undergraduate students at Double First-Class universities in China (18). The measure covers five domains: information (4 items), communication and collaboration (5 items), safety and privacy (7 items), digital content creation (3 items), and problem-solving (3 items). Items are rated from 1 (strongly disagree) to 5 (strongly agree), producing a total score of 22–110; higher scores indicate greater self-perceived general digital literacy. The score reflects broad self-perceptions in general digital environments rather than objectively demonstrated digital competence, digital health literacy, nursing informatics proficiency, or performance in clinical digital systems. Cronbach’s alpha was 0.832 in the development study and 0.869 in the present sample.

#### 2.3.3 Creative self-efficacy

Creative self-efficacy was assessed with a four-item Chinese adaptation based on the Creative Self-Efficacy Scale developed by Tierney and Farmer (10) and translated by Ma (19). Items are rated from 1 (strongly disagree) to 5 (strongly agree), giving a total score of 4–20; higher scores indicate greater confidence in performing creative tasks. The reported Cronbach’s alpha for the Chinese adaptation was 0.829, and Cronbach’s alpha in the present sample was 0.816.

#### 2.3.4 Innovative behavior

Innovative behavior was measured with a five-item Chinese adaptation of Scott and Bruce’s individual innovative behavior measure (5), revised by Liu and Shi (20). Items are rated from 1 (strongly disagree) to 5 (strongly agree), yielding a total score of 5–25; higher scores indicate stronger self-reported innovative-behavior tendencies. The scale captures students’ reports of idea generation, promotion, and application; it does not independently verify implementation, innovation quality or success, or resulting changes in practice and health outcomes. The reported Cronbach’s alpha for the Chinese adaptation was 0.865, and Cronbach’s alpha in the present sample was 0.811.

To strengthen measurement evidence beyond internal consistency, the three Chinese-language measures were evaluated jointly at the item level before the observed-total-score association analysis. Five theory-based confirmatory factor models were compared, and the evaluation reported standardized loadings, composite reliability, average variance extracted, standardized latent correlations, the Fornell–Larcker criterion, and HTMT with 5,000-resample bootstrap confidence intervals (21). This approach allowed the study to evaluate whether the score structure was consistent with the proposed three constructs and to identify any specific area of construct overlap rather than relying on Cronbach’s alpha alone. The procedures are specified in Section 2.5, and the results are reported in Section 3.3 and Tables 1–3. Because the study was not designed as a formal cross-cultural adaptation or measurement-invariance investigation, these results are interpreted as sample-specific psychometric evidence rather than definitive validation across populations.

**Table 1:** Confirmatory factor analysis model-fit comparisons.

Model	χ^2^	df	CFI	TLI	RMSEA (90% CI)	SRMR
M1: Simple three-factor	3072.17	431	0.806	0.790	0.111 (0.107–0.115)	0.090
M2: Simple two-factor (CSE + IB)	3224.10	433	0.795	0.779	0.114 (0.110–0.117)	0.090
M3: One-factor	5551.95	434	0.623	0.596	0.154 (0.150–0.158)	0.123
M4: Higher-order DL + separate CSE and IB	1314.15	426	0.935	0.929	0.065 (0.061–0.069)	0.068
M5: Higher-order DL + combined CSE/IB	1465.92	428	0.924	0.917	0.070 (0.066–0.074)	0.068

DL, digital literacy; CSE, creative self-efficacy; IB, innovative behavior; CFI, comparative fit index; TLI, Tucker–Lewis index; RMSEA, root mean square error of approximation; SRMR, standardized root mean square residual. M4 and M5 represented the 22 digital-literacy items with five first-order domain factors loading on a higher-order DL factor. M4 fitted significantly better than M5 (Δχ^2^ = 151.76, Δdf = 2, *p* < 0.001). No items were deleted and no *post hoc* residual correlations were added.

**Table 2:** Composite reliability and convergent-validity indices for the preferred higher-order three-construct model.

Construct	Loadings used for CR/AVE	CR	AVE	√AVE
Digital literacy (higher-order)	0.637–0.962	0.918	0.694	0.833
Creative self-efficacy	0.809–0.888	0.916	0.733	0.856
Innovative behavior	0.822–0.881	0.929	0.725	0.851

For digital literacy, the reported loading range refers to the five first-order domains loading on the higher-order construct; for creative self-efficacy and innovative behavior, it refers to standardized item loadings. CR, composite reliability; AVE, average variance extracted; √AVE, square root of AVE.

**Table 3:** Pairwise discriminant-validity evidence for the preferred higher-order three-construct model.

Construct pair	Latent *r*	HTMT	HTMT bootstrap 95% CI	Fornell-Larcker
DL-CSE	0.630	0.628	0.552–0.696	Met
DL-IB	0.631	0.628	0.548–0.697	Met
CSE-IB	0.907	0.902	0.858–0.941	Not met

DL, digital literacy; CSE, creative self-efficacy; IB, innovative behavior; latent r, standardized latent correlation; HTMT, heterotrait-monotrait ratio. Latent correlations were estimated in M4; HTMT confidence intervals were based on 5,000 bootstrap resamples. The Fornell–Larcker criterion was considered met when each construct’s √AVE exceeded the absolute latent correlation for that pair.

### 2.4 Data collection and quality control

Data were collected through Wenjuanxing, a Chinese online survey platform. After permission had been obtained from the participating nursing schools, class advisers distributed the survey link and QR code through class communication groups rather than posting the questionnaire on a public website. This targeted institution- and class-based approach directed recruitment toward the intended undergraduate nursing-student population, broadened the institutional sources represented beyond a single campus, and avoided unrestricted public access. The landing page described the study purpose, voluntary participation, confidentiality protections, and the right to discontinue. Participants indicated electronic consent before proceeding. Eligibility was assessed by self-report during online screening, and the analytic dataset contained only respondents who met the stated criteria. Required-response settings and logic checks were used for the retained questionnaire items, and the platform restricted each internet protocol address to one submission. Together, targeted distribution, electronic consent, required-response settings, logic checks, and the IP-based restriction created a structured survey pathway that supported questionnaire completeness and reduced the opportunity for repeat submissions; however, the IP-based restriction did not establish person-level uniqueness across different devices or networks. Responses were recorded directly in the survey platform, and completion required approximately 10–15 min. The available survey export and study records did not retain the number of students who received the invitation, opened the landing page, or consented. Consequently, the reconstructable participant flow began with the 508 post-screening records available for quality assessment. A conventional response rate and CHERRIES participation and completion rates could not be reconstructed; a website view rate was not applicable because the questionnaire was not hosted through a public website. Because distribution occurred within institutions and classes, observations may nevertheless have been clustered at either level. Institution and class identifiers and institution-specific sample sizes were not retained in the analytic dataset available for this report, precluding estimation of intraclass correlations, design effects, cluster-robust standard errors, or multilevel sensitivity analyses. Procedural safeguards relevant to response quality included the confidentiality assurance, voluntary participation, and the right to discontinue; these measures were intended to reduce evaluation apprehension and perceived pressure. They complemented the required-response settings, logic checks, IP-based submission restriction, and uniform record-level quality control by addressing the conditions under which students responded as well as the completeness of submitted records. Because the three focal scales were still completed in the same questionnaire session and the study did not include a social-desirability scale or a theoretically unrelated marker variable, socially desirable and other same-source response processes could not be fully excluded.

All records available in the survey export after online eligibility screening were evaluated before analysis using three uniform record-level quality-control criteria: incomplete questionnaire data, completion time of less than 300 s, or selection of the same response option for every Likert-scale item. Records meeting at least one criterion were excluded. Reason-specific counts and overlap among these criteria were not retained, so only the total number of post-screening quality-control exclusions can be reported. Because required-response settings were used for the retained items and incomplete records were excluded, the analytic dataset contained no item-level missing values. No nonresponse weighting or statistical correction for self-selection was undertaken because the recruitment denominator and characteristics of nonparticipants were unavailable.

### 2.5 Statistical analysis

Descriptive, correlational, product-of-coefficients, supplementary reverse-order, and Harman single-factor analyses were performed using IBM SPSS Statistics, version 27.0; confirmatory factor analyses were conducted in Python using semopy, version 2.3.11. Categorical variables were summarized as frequencies and percentages, and continuous variables as means and standard deviations. Internal consistency and construct validity were treated as complementary but distinct aspects of measurement quality. Cronbach’s alpha was reported for each scale, while dimensionality, convergent validity, and construct distinctiveness were evaluated separately through the confirmatory-factor-analysis procedures described below. Pearson correlation coefficients were calculated for digital literacy, creative self-efficacy, and innovative behavior.

Before the observed-total-score product-of-coefficients analysis, item-level confirmatory factor analyses compared five measurement models. M1 specified digital literacy, creative self-efficacy, and innovative behavior as three separate first-order factors. M2 combined creative self-efficacy and innovative behavior while retaining digital literacy as a separate factor, and M3 placed all 31 items on one factor. Because the digital-literacy measure contains five prespecified domains, M4 represented its 22 items by five first-order domain factors loading on a higher-order digital-literacy factor, with creative self-efficacy and innovative behavior retained as separate factors. M5 used the same higher-order digital-literacy structure but combined creative self-efficacy and innovative behavior. Item-level skewness ranged from −0.768 to −0.089 and excess kurtosis from −0.745 to 1.093; models were estimated by maximum likelihood using the Wishart objective. Model fit was evaluated using χ^2^, degrees of freedom, the comparative fit index (CFI), Tucker-Lewis index (TLI), root mean square error of approximation (RMSEA) with its 90% CI, and standardized root mean square residual (SRMR), together with the nested χ^2^ difference test for M4 versus M5. No items were deleted and no *post hoc* residual correlations were added. Convergent validity was evaluated using standardized loadings, composite reliability (CR), and average variance extracted (AVE). Discriminant validity was evaluated using standardized latent correlations from the preferred measurement model, the Fornell-Larcker criterion, and the heterotrait-monotrait ratio (HTMT) with 5,000-resample bootstrap CIs (21).

The primary cross-sectional product-of-coefficients analysis used PROCESS macro Model 4 (22), with digital literacy specified as the predictor, creative self-efficacy as the prespecified intermediate variable for the statistical decomposition, and innovative behavior as the outcome. This theory-informed ordering was specified to quantify the proposed association pattern and was not treated as evidence that creative self-efficacy temporally or causally intervened between the other two variables. The revised eight-variable set was not prespecified in the original protocol and is therefore described as a *post hoc*, theory-informed adjustment set. It comprised year of study, rural or urban residence, maternal educational attainment, student leadership experience, research-project participation, competition awards, proficiency certificates, and extracurricular practice. Selection followed substantive and temporal reasoning rather than automatic retention by a univariable *p*-value threshold (23). Year of study represented cumulative curricular, clinical, and digital-learning exposure (6, 7); residence and maternal education represented pre-university socioeconomic, informational, and digital-resource context (24); and the remaining variables represented prior learning engagement, mastery or achievement experiences, and exposure to diverse academic and practice settings. This grouping is consistent with social cognitive theory (9, 10) and evidence linking diversity and practice experiences to creative self-efficacy and innovative behavior in medical and nursing students (11, 12). All eight covariates were entered in both regression equations. The adjustment was designed to evaluate whether the focal association pattern remained evident after accounting for these measured demographic and educational differences, thereby strengthening interpretation beyond an unadjusted specification. Because the temporal precedence of all covariates cannot be verified in a cross-sectional survey, the resulting coefficients are interpreted as conditional associations involving theory-informed covariates rather than independent causal effects.

The covariate-adjusted model was designated as the primary analysis, while the unadjusted model was retained as a sensitivity analysis so that dependence on the specified adjustment set could be evaluated directly. The product-of-coefficients component involving creative self-efficacy was estimated from 5,000 participant-level bootstrap resamples; a 95% confidence interval (CI) that did not include zero was interpreted as evidence that this statistical product term differed from zero. To examine whether the contemporaneous correlation structure uniquely supported the prespecified ordering, a supplementary *post hoc* reverse-order calculation interchanged creative self-efficacy and innovative behavior while retaining digital literacy as the predictor. Standardized coefficients for the alternative ordering digital literacy → innovative behavior → creative self-efficacy and their product were calculated algebraically from the observed-score correlation matrix. Because either three-variable ordering with a direct path is a saturated reparameterization of the same covariance structure, comparative global fit cannot select between them. This calculation was therefore used only as a directional-nonuniqueness check; no bootstrap, temporal, or causal inference was attached to it. Tests were two-sided, with *p* < 0.05 considered statistically significant. Given the cross-sectional design, all coefficients were interpreted as associations and the product term as a statistical decomposition of contemporaneous covariance, not as evidence of a temporal or causal mechanism (25). PROCESS used observed scale totals and was not a latent-variable structural model. Because institution and class identifiers were unavailable, the bootstrap resampled students as independent observations and did not model potential recruitment clusters. The reported standard errors and confidence intervals therefore describe precision under the individual-level analysis and may be narrower than cluster-aware intervals if responses were positively correlated within institutions or classes.

Common method variance was examined *post hoc* using two complementary diagnostics. First, all 31 focal items were entered into an unrotated principal-components analysis, and the percentage of total item variance accounted for by the first component was recorded. This Harman analysis was treated as a supplemental diagnostic rather than a decisive test. Second, the single-factor CFA (M3) was compared with the simple three-factor model (M1) and the theory-consistent higher-order three-construct model (M4), providing a more informative assessment of whether one general factor could adequately reproduce the item covariance. The diagnostics were interpreted together with the study design: evidence against a single dominant factor does not quantify or exclude more diffuse common method variance (26).

### 2.6 Ethical considerations

The study protocol, including the eligibility criteria, was reviewed and approved by the Ethics Committee of the First Affiliated Hospital of Wannan Medical University (Yijishan Hospital of Wannan Medical University), Wuhu, China (Research Ethics Review No. 189 [2025]).

Before accessing the questionnaire, participants viewed an electronic information sheet describing the study purpose, procedures, voluntary nature of participation, confidentiality protections, and right to discontinue, and provided electronic informed consent. The mental-health screening item concerned sensitive personal information, relied on self-report, and was used only for eligibility screening rather than to establish a study diagnosis or clinical assessment. The information sheet emphasized confidentiality, voluntariness, and the right to discontinue. The criterion was not intended to imply that affected students lacked the capacity to consent or provide valid responses; its proportionality and potential stigma-related implications are addressed in the limitations.

## 3 Results

### 3.1 Participant flow and characteristics

After online eligibility screening, 508 records were available in the survey export for quality assessment. Following uniform post-screening quality control, 502 records (98.8%) were retained for analysis, indicating limited record loss once responses entered this assessment stage. The six excluded records met at least one quality-control criterion because they contained incomplete questionnaire data, were completed in less than 300 s, or contained the same response option for every Likert-scale item. Reason-specific counts and overlap among these criteria were not retained. The 98.8% value is a post-screening record-retention indicator rather than a conventional response rate, participation rate, or CHERRIES completion rate. The available institutional and platform records did not provide the number of students invited, landing-page visitors, consenters, or students screened out under each eligibility criterion. Consequently, a complete invited-opened-consented-screened-eligible flow and the corresponding survey uptake metrics could not be reconstructed.

Among the 502 participants, 416 (82.9%) were female and 86 (17.1%) were male. Students from all four study years were represented: 141 (28.1%) were in the first year, 176 (35.1%) in the second year, 168 (33.5%) in the third year, and 17 (3.4%) in the fourth year. The first- to third-year groups together comprised 485 participants (96.6%), providing substantial representation across the first three undergraduate years, whereas representation of the fourth year was limited. A total of 248 participants (49.4%) reported rural residence and 254 (50.6%) urban residence.

### 3.2 Descriptive statistics and correlations

The mean (SD) scores were 82.22 (14.06) for self-perceived general digital literacy, 14.29 (2.72) for creative self-efficacy, and 17.67 (3.39) for self-reported innovative behavior. Self-perceived general digital literacy was positively correlated with creative self-efficacy (*r* = 0.574, *p* < 0.001) and self-reported innovative behavior (*r* = 0.584, *p* < 0.001). Creative self-efficacy was strongly and positively correlated with self-reported innovative behavior (*r* = 0.834, *p* < 0.001; Table 4).

**Table 4:** Scale properties, descriptive statistics, and Pearson correlations (*n* = 502).

Variable	Possible range	Mean (SD)	Cronbach’s α	1	2	3
1. Digital literacy	22–110	82.22 (14.06)	0.869	1		
2. Creative self-efficacy	4–20	14.29 (2.72)	0.816	0.574***	1	
3. Innovative behavior	5–25	17.67 (3.39)	0.811	0.584***	0.834***	1

***p < 0.001. Scores are self-reported total scores. Digital literacy denotes self-perceived general digital literacy, and innovative behavior denotes self-reported behavioral tendencies. Cronbach’s alpha summarizes within-scale internal consistency only and does not establish unidimensionality, convergent validity, or discriminant validity; measurement-model and construct-validity results are reported in Tables 1–3.

### 3.3 Measurement structure, construct validity, and common-method diagnostics

The simple three-factor model (M1) did not fit the data satisfactorily (χ^2^ = 3072.17, df = 431, CFI = 0.806, TLI = 0.790, RMSEA = 0.111, 90% CI, 0.107–0.115; SRMR = 0.090), although it fitted better than both the simple two-factor model combining creative self-efficacy with innovative behavior (M2: χ^2^ = 3224.10, df = 433, CFI = 0.795, TLI = 0.779, RMSEA = 0.114, 90% CI, 0.110–0.117; SRMR = 0.090) and the one-factor model (M3: χ^2^ = 5551.95, df = 434, CFI = 0.623, TLI = 0.596, RMSEA = 0.154, 90% CI, 0.150–0.158; SRMR = 0.123). After the five prespecified digital-literacy domains were represented as first-order factors, the theory-consistent higher-order three-construct model (M4) showed acceptable fit (χ^2^ = 1314.15, df = 426, CFI = 0.935, TLI = 0.929, RMSEA = 0.065, 90% CI, 0.061–0.069; SRMR = 0.068). M4 fitted significantly better than the corresponding higher-order model combining creative self-efficacy and innovative behavior (M5: χ^2^ = 1465.92, df = 428, CFI = 0.924, TLI = 0.917, RMSEA = 0.070, 90% CI, 0.066–0.074; SRMR = 0.068; Δχ^2^ = 151.76, Δdf = 2, *p* < 0.001; Table 1).

In M4, the standardized loadings used to define the higher-order digital-literacy construct ranged from 0.637 to 0.962; the corresponding ranges were 0.809–0.888 for creative self-efficacy and 0.822–0.881 for innovative behavior. CR values were 0.918, 0.916, and 0.929, and AVE values were 0.694, 0.733, and 0.725, respectively (Table 2). The digital literacy-creative self-efficacy and digital literacy-innovative behavior pairs met the Fornell-Larcker criterion and had HTMT values of 0.628 (95% CI, 0.552–0.696) and 0.628 (95% CI, 0.548–0.697), respectively. In contrast, the standardized latent correlation between creative self-efficacy and innovative behavior in M4 was 0.907, and their HTMT was 0.902 (95% CI, 0.858–0.941). The square roots of AVE for creative self-efficacy (0.856) and innovative behavior (0.851) were both below their latent correlation, so the Fornell-Larcker criterion was not met for this pair (Table 3). Taken together, the acceptable model fit, high CR and AVE values, and discriminant-validity evidence for both pairs involving digital literacy supported the three-construct measurement representation overall. The only less clearly resolved boundary was between creative self-efficacy and innovative behavior: the comparative models favored retaining them as separate factors, although strict discriminant validity for this pair was not fully established.

In the supplemental unrotated Harman analysis, four components had eigenvalues greater than 1, and the first component accounted for 49.47% of total item variance. Consistent with the multidimensional measurement structure, the one-factor CFA fitted poorly (M3: χ^2^ = 5551.95, df = 434, CFI = 0.623, TLI = 0.596, RMSEA = 0.154, 90% CI, 0.150–0.158; SRMR = 0.123) and markedly worse than M4. Together, these results indicate that the covariance among the 31 items was not adequately represented by a single general factor. Because *post hoc* diagnostics cannot quantify all method-related covariance, they do not rule out more diffuse common method variance arising from the same-source, same-time self-report design.

### 3.4 Covariate-adjusted association decomposition involving creative self-efficacy

In the covariate-adjusted primary model, the self-perceived general digital-literacy score was positively associated with creative self-efficacy (B = 0.114, β = 0.565, t = 15.274, *p* < 0.001). When the digital-literacy score, creative self-efficacy, and the eight covariates were included in the outcome equation, creative self-efficacy was positively associated with the self-reported innovative-behavior score (B = 0.920, β = 0.740, t = 24.222, *p* < 0.001). The adjusted total coefficient relating self-perceived general digital literacy to self-reported innovative behavior was also statistically different from zero (B = 0.145, β = 0.579, t = 15.619, *p* < 0.001; Table 5; Figure 1). Reporting B and β together for the prespecified component paths and total association makes both the scale-specific magnitude and the standardized magnitude of the primary associations directly assessable.

**Table 5:** Regression coefficients for the prespecified covariate-adjusted cross-sectional decomposition (*n* = 502).

Path	*B*	*β*	*t*	*p*
Digital literacy → creative self-efficacy	0.114	0.565	15.274	<0.001
Creative self-efficacy → innovative behavior	0.920	0.740	24.222	<0.001
Digital literacy → innovative behavior (total coefficient)	0.145	0.579	15.619	<0.001

B, unstandardized coefficient; β, standardized coefficient. The prespecified a, b, and total-association (c) coefficients are shown in both metrics, allowing the paths to be interpreted in their original units and compared on a standardized scale. The adjusted direct coefficient (c′ = 0.040; SE = 0.008; 95% CI, 0.025–0.055) and the bootstrap product term (B = 0.105; SE = 0.010; 95% CI, 0.087–0.124) are reported in Table 6 and Figure 1. All regression equations included the eight specified covariates. Variable names denote self-report scale totals: digital literacy is self-perceived general digital literacy, and innovative behavior is self-reported. The complete coefficient set is internally consistent across the results, Figure 1; Tables 5, 6. The arrows and coefficient labels denote the theory-informed statistical ordering only and do not establish temporal sequence. These observed-total-score coefficients should be interpreted together with the supplementary directional check and the measurement-validity evidence reported in Tables 1–3.

**Figure 1:**
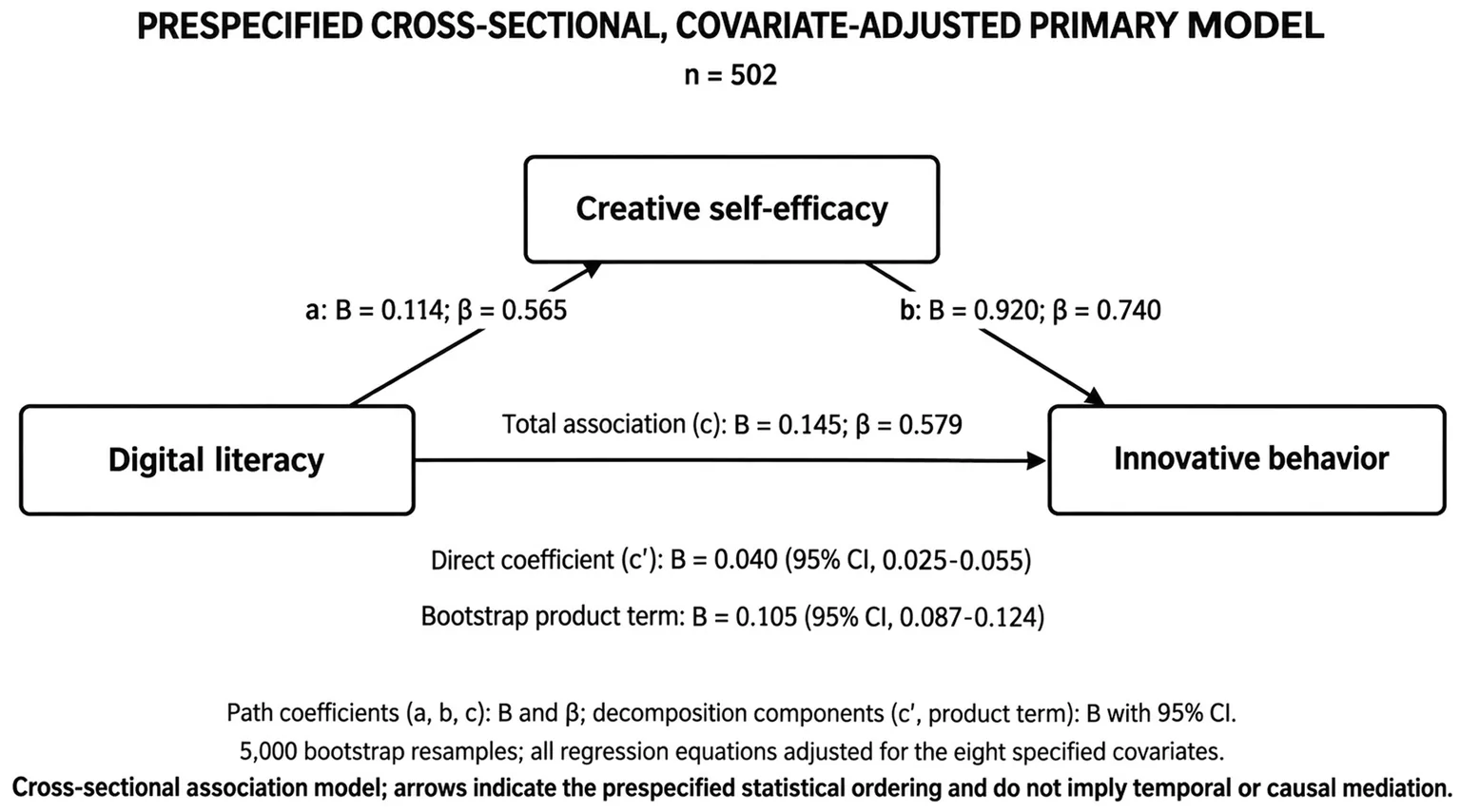
Visual synthesis of the prespecified cross-sectional, covariate-adjusted primary product-of-coefficients model (*n* = 502). Unstandardized coefficients (*B*) and standardized coefficients (*β*) are shown together for the *a* path, *b* path, and total association (*c*), permitting evaluation in both the original scale units and standardized units. The adjusted direct coefficient was *B* = 0.040 (*SE* = 0.008; 95% *CI*, 0.025–0.055), and the bootstrap product term involving creative self-efficacy was *B* = 0.105 (*SE* = 0.010; 95% *CI*, 0.087–0.124; 5,000 bootstrap resamples). The conventional construct labels denote the measured self-report totals: self-perceived general digital literacy and self-reported innovative behavior. All regression equations adjusted for year of study, rural or urban residence, maternal educational attainment, student leadership experience, research-project participation, competition awards, proficiency certificates, and participation in extracurricular practice activities. Together, the sample size, dual coefficient reporting, bootstrap precision, and explicit covariate status provide a compact visual summary that can be cross-checked directly against Tables 5, 6. The arrows indicate the prespecified statistical ordering in contemporaneous data and do not imply temporal or causal mediation.

The adjusted total coefficient for the association between self-perceived general digital literacy and self-reported innovative behavior was 0.145 (SE = 0.009, 95% CI, 0.127–0.163). After creative self-efficacy was included, the adjusted direct coefficient was 0.040 (SE = 0.008, 95% CI, 0.025–0.055), and the bootstrap product-of-coefficients component involving creative self-efficacy was 0.105 (SE = 0.010, 95% CI, 0.087–0.124). The product-term interval excluded zero, had an absolute width of 0.037, and had an approximate half-width of 0.019, equivalent to approximately 17.6% of the point estimate. The adjusted and unadjusted product-term estimates were also materially similar. Together, the bootstrap precision and cross-specification consistency provide complementary support for a stable association pattern among the three scale scores and identify creative self-efficacy as a theoretically relevant correlate for prospective investigation. Within the cross-sectional design, the product term is a decomposition of contemporaneous self-report associations rather than a temporal or causal mediation effect.

**Table 6:** Total, direct, and product-of-coefficients components in the covariate-adjusted cross-sectional model.

Coefficient	Estimate	SE	95% CI
Total coefficient	0.145	0.009	0.127–0.163
Direct coefficient	0.040	0.008	0.025–0.055
Product term involving creative self-efficacy	0.105	0.010	0.087–0.124

The product-of-coefficients component involving creative self-efficacy was estimated from 5,000 participant-level bootstrap resamples. CI, confidence interval; SE, standard error. Its bootstrap 95% CI had an absolute width of 0.037 and an approximate half-width of 0.019 (approximately 17.6% of the point estimate). Together with the analytic sample of 502 and the close adjusted and unadjusted estimates, this interval provides a direct description of empirical precision and cross-specification stability under the individual-level analysis. Institution and class identifiers were unavailable, so potential clustering could not be incorporated. The model adjusted for year of study, rural or urban residence, maternal educational attainment, student leadership experience, research-project participation, competition awards, proficiency certificates, and participation in extracurricular practice activities. Coefficients are based on self-report scale totals. The product term is a statistical component of a contemporaneous association and should not be interpreted as the proportion of innovative behavior explained or caused by creative self-efficacy or as evidence of temporal or causal mediation.

### 3.5 Covariate sensitivity and supplementary directional check

The unadjusted model produced a closely similar prespecified decomposition. Digital literacy was associated with creative self-efficacy (B = 0.111, β = 0.575, *p* < 0.001), and creative self-efficacy was associated with innovative behavior after digital literacy was included (B = 0.925, β = 0.743, *p* < 0.001). The unadjusted total, direct, and product-of-coefficients estimates were 0.141 (95% CI, 0.124–0.158), 0.038 (95% CI, 0.024–0.052), and 0.103 (95% CI, 0.085–0.122), respectively. Their closeness to the adjusted estimates reinforces the stability of the principal statistical finding across the two specifications, although residual and unmeasured confounding cannot be excluded. In the supplementary *post hoc* check calculated from the same observed-score correlation matrix, the reverse ordering digital literacy → innovative behavior → creative self-efficacy yielded a standardized product of approximately 0.442, compared with approximately 0.427 (0.575 × 0.743) in the prespecified unadjusted ordering. The positive product under both parameterizations indicates that the observed association pattern is not dependent on only one variable ordering, while also showing that these cross-sectional data cannot determine the temporal direction. No bootstrap or causal inference was attached to this descriptive calculation.

## 4 Discussion

### 4.1 Principal findings

In this pooled multi-institutional sample of 502 undergraduate nursing students, higher self-perceived general digital literacy was associated with stronger creative self-efficacy and more self-reported innovative behavior. The measurement analyses provided important support for the integrity of the three-construct representation: the theory-consistent higher-order model achieved acceptable fit, significantly outperformed the corresponding model combining creative self-efficacy and innovative behavior, and fitted markedly better than the one-factor alternative. The common-method diagnostics likewise showed that the item covariance could not be reduced to a single general factor. In the covariate-adjusted observed-score model, the bootstrap interval for the prespecified product-of-coefficients component excluded zero, a smaller direct coefficient for digital literacy remained, and adjusted and unadjusted estimates were materially similar. These converging results support a coherent and statistically stable association pattern across measurement-model comparisons and covariate specifications. Substantively, the study connects two lines of evidence previously examined separately among nursing students: self-perceived digital literacy in relation to self-reported innovative behavior (7) and creative self-efficacy in relation to innovative behavior within a clinical-practice-environment model (11). Examining all three constructs together demonstrates that the complete pattern is observable in a pooled nursing-student sample recruited from three institutions, extending the scope of earlier medical-student evidence (12) without claiming profession-level differences. Multi-institutional recruitment broadened the institutional sources represented beyond one campus. The high creative self-efficacy-innovative behavior latent correlation, borderline HTMT value, and failure of the Fornell-Larcker criterion mean that their empirical boundary was not fully resolved; nevertheless, the comparative evidence favored retaining three constructs over the plausible combined and one-factor representations. Within this clearly defined inferential scope, the study provides a rigorously characterized and nursing-relevant relationship among student-reported perceptions, efficacy beliefs, and behavioral tendencies.

### 4.2 Digital literacy and innovative behavior

The positive association between self-perceived general digital literacy and self-reported innovative behavior is consistent with a latent-profile analysis of Chinese undergraduate nursing students (7), configurational evidence among medical students (8), and the medical-student indirect-effect study noted above (12). The nursing latent-profile study showed that students with higher digital-literacy profiles reported more innovative behavior; the present study extends that nursing evidence by evaluating creative self-efficacy within the same three-variable model. At the level of the actual measures, students who rated themselves more highly on locating, evaluating, creating, communicating, and applying information in digital environments also tended to report stronger engagement in generating, promoting, or applying ideas. Evidence appraisal, information integration, communication, and digital creation offer plausible educational explanations for this pattern. However, these processes were not observed directly, and the association may also reflect academic agency, motivation, prior research exposure, institutional resources, reciprocal self-perceptions, or same-source measurement. The cross-sectional design cannot distinguish among these explanations. The finding therefore supplies a focused and theoretically grounded hypothesis for prospective studies that pair self-report scales with objective digital tasks and independently assessed innovative outputs.

### 4.3 Creative self-efficacy in the cross-sectional association pattern

The adjusted product-of-coefficients component was B = 0.105 (95% CI, 0.087–0.124), compared with a total coefficient of B = 0.145 and a direct coefficient of B = 0.040. In observed-scale units, the product of the two fitted component coefficients corresponds to a 0.105-point component in the expected self-reported innovative-behavior score per one-point difference in self-perceived general digital literacy, conditional on the eight measured covariates. This number is scale-, specification-, and ordering-dependent. It is not a standalone effect-size index or the proportion of innovative behavior explained or caused by creative self-efficacy; the product-to-total ratio is not interpreted as a mediated or causal proportion, and the smaller direct coefficient does not establish partial mediation. The product term is therefore only a descriptive decomposition of contemporaneous covariance. Within this noncausal scope, the pattern is compatible with social cognitive theory, which emphasizes the relevance of efficacy beliefs to effort, persistence, and behavioral choice (9, 10). This relationship has particular educational relevance in nursing because students learn to use digital information and technologies while developing evidence-informed judgment, communicating within care teams, and proposing improvements under clinical supervision, professional accountability, and patient-safety expectations (6, 27–30). These nursing-specific demands justify examining the constructs together. Previous nursing evidence identified an indirect association involving the clinical practice environment, creative self-efficacy, and innovative behavior but did not include digital literacy (11). The present study therefore makes an integrative contribution by identifying creative self-efficacy as a theory-informed correlate connecting two previously separate nursing-education evidence streams and as *a priori*ty for longitudinal and intervention evaluation, without implying that the present scales assessed clinical performance.

The measurement findings provide affirmative evidence for the proposed three-construct framework. Across five item-level CFA models, the theory-consistent higher-order model achieved acceptable fit, markedly outperformed the one-factor model, and significantly outperformed the model combining creative self-efficacy and innovative behavior (Δχ^2^ = 151.76, Δdf = 2, *p* < 0.001). High standardized loadings together with CR and AVE values supported the reliability and convergent validity of the specified factors, whereas the superior three-construct fit argued against treating creative self-efficacy and innovative behavior as wholly redundant. Distinctiveness was evaluated separately: their latent correlation was 0.907, HTMT was 0.902 (95% CI, 0.858–0.941), and the Fornell-Larcker criterion was not met. The comparative evidence therefore supports retaining related but non-equivalent constructs, while strict discriminant validity was not fully established and item-content overlap or shared response processes may inflate the observed-score product term (21, 26). The poor one-factor fit and close adjusted and unadjusted estimates strengthen confidence in the overall cross-sectional pattern but do not resolve this boundary or establish directionality. Future research should cross-validate the competing CFA structures, repeat HTMT assessment with bootstrap confidence intervals and the Fornell-Larcker criterion, test measurement invariance across sex, year of study, and institution, and use expert item mapping and cognitive interviews. If distinctiveness is replicated, latent-variable models with temporally separated or independently assessed outcomes should follow (21, 25).

### 4.4 Implications for prospective nursing-education research and evaluation

The findings provide an empirically grounded rationale for evaluating self-perceived digital literacy and creative self-efficacy together rather than treating them as unrelated educational targets. A prospective curriculum hypothesis is that learning designs combining digital-information practices with opportunities for mastery, modeling, feedback, and creative problem-solving may strengthen both students’ perceived capabilities and their engagement with innovative activities. Candidate components include guided appraisal of online evidence, nursing informatics and data interpretation, responsible use of artificial intelligence, privacy and cybersecurity, authentic problem-based projects, simulation, research or quality-improvement participation, peer modeling, mentorship, and formative feedback. Digital-competency toolkits and online digital-health and informatics courses illustrate feasible content for adaptation and evaluation (27, 28), while a recent scoping review identified substantial variation and continuing gaps in digital-health education across nursing programs (29). A curriculum-development report from nursing education in China likewise illustrates how stepped coursework, adaptive digital resources, research-driven practice, and ethics can be integrated (30). These examples translate the observed association pattern into testable educational directions; the present cross-sectional study does not determine which component or combination is effective. Because previous nursing evidence identifies the clinical practice environment as relevant to creative self-efficacy and innovative behavior (11), future studies should also measure that environment directly.

A rigorous prospective evaluation should prespecify the intervention, comparator, follow-up points, and outcomes and distinguish proximal changes in perceptions and efficacy beliefs from demonstrated performance. A layered outcome framework could include the present self-report measures; objective digital-information tasks; assessments of digital health literacy or nursing informatics proficiency; performance in simulated or authentic clinical digital systems; and independently rated or verified innovation proposals, implementation behaviors, or project outputs. This design would allow investigators to test whether changes in the student-reported constructs precede and predict objectively assessed educational or practice-relevant outcomes. Claims about digitally enabled clinical readiness, public health performance, patient care improvement, or successful innovation require those outcomes to be measured directly. Thus, the current findings offer a clear rationale and measurable hypotheses for prospective nursing-specific curriculum evaluation rather than evidence that a particular intervention already improves practice.

### 4.5 Strengths and limitations

This study has several substantive, procedural, analytic, and reporting strengths. It jointly evaluated self-perceived general digital literacy, creative self-efficacy, and self-reported innovative behavior in a comparatively large pooled three-institution nursing-student sample, connecting two lines of nursing evidence that had previously been examined separately. Recruitment from three institutions broadened the institutional sources represented within Anhui Province and reduced dependence on a single-campus context. The final analytic sample of 502 was 82.5% larger than the protocol-defined target, corresponded to 22.8 respondents per item for the 22-item primary measure, and retained 502 of 508 post-screening records (98.8%) after uniform quality control; the analytic dataset contained no item-level missing values. These features provided a strong empirical base for the item-level measurement analyses and observed-score association models. Measurement evaluation extended well beyond Cronbach’s alpha. The theory-consistent higher-order three-construct model achieved acceptable fit, significantly outperformed the corresponding model combining creative self-efficacy and innovative behavior, and fitted markedly better than the one-factor alternative. High standardized loadings, CR values of 0.918, 0.916, and 0.929, and AVE values of 0.694, 0.733, and 0.725 provided strong reliability and convergent-validity evidence for the three factors. Construct distinctiveness was evaluated separately: both construct pairs involving digital literacy met the Fornell-Larcker criterion and had HTMT values of 0.628, while the better fit of the three-construct model supported retaining creative self-efficacy and innovative behavior as related but non-equivalent factors despite their less fully resolved boundary. The analytic strategy strengthened interpretation beyond an unadjusted specification by designating the eight-covariate model as primary, using 5,000-resample bootstrap estimation, and retaining the unadjusted model as a sensitivity analysis. The adjusted and unadjusted product-term estimates were closely similar (0.105 and 0.103), and the adjusted bootstrap CI had an approximate half-width of 0.019. This cross-specification consistency supports the empirical stability and useful precision of the principal pattern under the individual-level analysis without converting the coefficients into independent or causal effects. Online implementation used targeted class-group distribution, electronic consent, required-response settings, logic checks, an IP-based submission restriction, and uniform post-screening quality-control criteria. The study also triangulated Harman’s supplemental diagnostic with alternative CFA models, conducted a reverse-order directional check, and presented a Figure 1 that integrates sample size, unstandardized and standardized coefficients, the bootstrap product-term CI, resampling details, and covariate status for direct cross-checking against Tables 5, 6. This combination of multi-institutional breadth, high record retention, comprehensive measurement evaluation, covariate sensitivity, bootstrap precision, directional transparency, and internally consistent reporting strengthens the credibility and usefulness of the pooled-sample findings.

The study’s limitations delimit external and causal inference but do not negate the internally coherent findings in the pooled sample. First, the cross-sectional design does not establish temporal sequence or causal mediation (25). The supplementary reverse-order calculation appropriately limits directional inference, while the adjusted and unadjusted results still demonstrate a stable contemporaneous association pattern. Second, convenience sampling within one Chinese province means that the findings cannot be assumed to represent all nursing students. Recruitment from three institutions nevertheless broadened the institutional sources represented beyond a single campus. Because institution- and class-level identifiers, institution-specific sample sizes, and cluster sizes were unavailable, intraclass correlations, cluster-robust standard errors, and multilevel sensitivity analyses could not be estimated. The individual-level analysis therefore treated the 502 students as independent, and the participant-level bootstrap did not correct for clustering. If responses were positively correlated within institutions or classes, the reported standard errors may have been underestimated and the confidence intervals narrowed. This limitation chiefly constrains confidence in the reported precision; the point estimates continue to describe the pooled sample under the individual-level model. Post-screening participant flow and record-level data quality were documented transparently: 502 of 508 available records (98.8%) were retained after uniform quality control, and the analytic dataset contained no item-level missing values. These indicators support the completeness of the analyzed records but should be distinguished from recruitment representativeness. Because institutional invitation denominators and counts of landing-page visitors, consenters, and screened-out students were unavailable, a conventional response rate and the corresponding CHERRIES participation and completion rates could not be calculated. Voluntary recruitment through class communication groups may have disproportionately represented students with greater digital engagement, stronger interest in innovation, or more regular engagement with those groups; without denominator and nonparticipant data, the direction and magnitude of any resulting nonresponse or self-selection bias could not be assessed or addressed through weighting. The IP-based restriction reduced the opportunity for repeat submissions but did not verify person-level uniqueness across devices or networks. Third, the protocol’s transparent two-stage recruitment planning used a cited respondent-to-item heuristic and a prespecified 20% operational allowance rather than an a priori effect-size-based calculation for the product term. The available study documentation did not identify pilot data or a site-specific historical loss rate used to derive the exact 20% allowance, which should not be interpreted as a universal recommendation. Nevertheless, the achieved sample of 502 was 82.5% larger than the 275-participant target, corresponded to 22.8 respondents per item for the 22-item primary measure, approached the conservative published small-small mediation benchmark, exceeded the small-small-to-medium and medium-medium benchmarks, and yielded a bounded bootstrap interval for the primary product term. These retrospective findings support the sensitivity and empirical precision of the primary analysis but do not establish prospectively adequate precision for every regression or measurement-model parameter, particularly under unassessed clustering. Fourth, no medical-student comparison group was included, and differences in the magnitude or structure of the associations between nursing and medical students cannot be inferred. Fifth, designating the covariate-adjusted model as primary directly strengthened the analysis beyond reliance on an unadjusted specification, and the close adjusted and unadjusted estimates indicate that the focal pattern was not materially dependent on the eight measured covariates. However, the adjustment set was developed for the revised analysis rather than prespecified in the original protocol, and the cross-sectional questionnaire cannot verify that every experiential covariate preceded the focal variables. The set was also not exhaustive: prior digital training, academic motivation, institutional resources, and the clinical practice environment were not directly measured and may remain relevant. The adjusted model should therefore be viewed as a conditional robustness analysis rather than proof of independent effects; residual confounding and possible overadjustment remain. Sixth, the confidentiality procedures and complementary Harman/CFA diagnostics supported response quality and showed that a single general factor did not adequately reproduce the item covariance. Nevertheless, all focal variables were measured by self-report at the same time, and no temporal or source separation, social-desirability measure, marker variable, or objective or observer-rated outcome was available. Socially desirable responding and more diffuse common method variance therefore cannot be fully excluded (26). Seventh, the measurement analyses supported the three-construct representation overall: the preferred model achieved acceptable fit, CR and AVE values were high, both construct pairs involving digital literacy met the Fornell-Larcker criterion, and the preferred model outperformed the model combining creative self-efficacy and innovative behavior. The remaining concern was specific to the creative self-efficacy-innovative behavior pair, for which the latent correlation was 0.907, HTMT was 0.902, and the Fornell-Larcker criterion was not met. Strict discriminant validity for this pair was therefore not fully established, and the observed-total-score product term may partly reflect construct overlap or shared response processes (21). Eighth, the digital-literacy instrument assessed self-perceived general digital literacy rather than objective digital competence or nursing-specific digital-health performance, and the innovative-behavior scale assessed reported tendencies rather than independently verified implementation or outcomes. The findings therefore should not be treated as direct evidence of clinical readiness, patient or public health improvement, successful innovation, or curriculum effectiveness. The clinical practice environment was not measured, so its proposed relevance is derived from prior nursing evidence rather than a mechanism tested here. Ninth, the exclusions based on a recent major life event and self-reported mental or psychological disorder were prespecified in the ethics-approved protocol rather than introduced after inspection of the outcomes, which provides procedural transparency and reduces concern about data-driven selection. Prespecification, however, does not itself establish scientific necessity or eliminate selection and equity concerns. Their operationalization was non-standardized, no eligibility-screening log is available to provide reason-specific counts or permit comparison with included students, and the available study documentation does not provide a scientific rationale demonstrating that these students could not complete the questionnaire validly or explaining why less restrictive inclusion strategies were not chosen. Consequently, the criteria may have reduced representativeness and introduced unquantifiable selection bias. Students experiencing greater distress may also differ in creative self-efficacy, digital engagement, or innovative behavior, but the direction and magnitude of any bias cannot be determined because screened-out students are absent from the analytic data. The adjusted-versus-unadjusted sensitivity analysis among included participants does not address this selection process. Use of self-reported mental-health status for exclusion also raises concerns about proportionality, disclosure, and potential stigmatization. These concerns do not imply that excluded students lacked the capacity to consent or respond validly. Future questionnaire studies should avoid mental-health- or distress-based exclusion unless clearly justified, use standardized definitions, retain and report screening counts by reason, and preferably include such students while measuring relevant status for prespecified descriptive, adjusted, stratified, or sensitivity analyses, with appropriate support or referral information where needed. The pooled three-institution sample provided substantial representation of the first three undergraduate years, and year of study was included in both equations of the covariate-adjusted primary model. Together with the close adjusted and unadjusted estimates, these features support the stability of the principal association pattern within the achieved sample. Nevertheless, women comprised 82.9% of participants, and only 17 students (3.4%) were in the fourth year. The findings therefore primarily reflect female nursing students and students in years 1–3 and should not be assumed to represent male or fourth-year students with equivalent precision. Adjustment for year of study supports pooled estimation conditional on measured year differences but cannot substitute for adequately powered subgroup analysis. Future multi-institutional studies should consider stratified or quota-based recruitment and, when sufficiently powered, evaluate sex- and year-specific estimates and interaction effects. More broadly, longitudinal, multi-region, and harmonized comparative studies should prospectively specify effect-size assumptions and covariates; retain invitation rosters, platform visitor, consent, screening, and completion counts; use proportionate person-level duplicate controls; report applicable CHERRIES response metrics; retain institution and class identifiers; report cluster sizes; account for clustering in design and analysis; measure the clinical practice environment directly; test measurement invariance; replicate the measurement structure in independent samples; and incorporate objective digital tasks, nursing-informatics or clinical-system performance assessments, and independently rated or verified innovation outputs before testing causal pathways, curriculum effects, practice outcomes, or profession-level differences.

## 5 Conclusion

Among 502 undergraduate nursing students recruited from three institutions in Anhui Province, higher self-perceived general digital literacy was associated with stronger creative self-efficacy and more self-reported innovative behavior. The theory-consistent higher-order three-construct model fitted acceptably, markedly outperformed the one-factor model, and significantly outperformed the model combining creative self-efficacy and innovative behavior. The bootstrap interval for the prespecified product-of-coefficients component excluded zero, and the adjusted and unadjusted estimates were materially similar, supporting the statistical stability of the observed association pattern across the two specifications. By evaluating all three constructs within one nursing-student model, the study connects two previously separate lines of nursing evidence and extends the complete association pattern to a pooled sample with broader institutional representation than a single-campus study. Creative self-efficacy is therefore identified as a theoretically meaningful correlate and a priority construct for prospective nursing-education research. The contribution is a measurement-informed and analytically robust characterization of relationships among students’ digital-literacy perceptions, creative efficacy beliefs, and reported innovative behavior. Longitudinal and intervention studies incorporating objective digital-performance measures and independently assessed innovation outcomes are needed to determine whether and how these relationships translate into educational or practice-relevant change.

## Data Availability

The datasets presented in this study can be found in online repositories. The names of the repository/repositories and accession number(s) can be found in the article/supplementary material.
